# Selective Autophagy of Peroxisomes in Plants: From Housekeeping to Development and Stress Responses

**DOI:** 10.3389/fpls.2019.01021

**Published:** 2019-08-28

**Authors:** Adela Olmedilla, Luisa M. Sandalio

**Affiliations:** Department of Biochemistry and Molecular and Cellular Biology of Plants, Estación Experimental del Zaidín, Consejo Superior de Investigaciones Científicas (CSIC), Granada, Spain

**Keywords:** autophogy-related genes, peroxins, pexophagy, plants, reactive oxygen species

## Abstract

Peroxisomes are dynamic organelles involved in multiple functions, including oxygen and nitrogen reactive species metabolism. In plants, these organelles have a close relationship with chloroplasts and mitochondria, characterized by intense metabolic activity and signal transduction. Peroxisomes undergo rapid changes in size, morphology, and abundance depending on the plant development stage and environmental conditions. As peroxisomes are essential not only for redox homeostasis but also for sensing stress, signaling transduction, and cell survival, their formation and degradation need to be rigorously regulated. In this review, new insights into the regulation of plant peroxisomes are briefly described, with a particular emphasis on pexophagy components and their regulation.

## Introduction

Peroxisomes are small round organelles surrounded by a single lipid bilayer present in most eukaryotes (Sandalio and Romero-Puertas, 2015; Kao et al., 2018). Despite their morphological similarity and the conservation of their functions in all eukaryotes, major differences have been found between peroxisomes in plants and peroxisomes in fungi and animals (Kamada et al., 2003). Initially, peroxisomes were regarded as H_2_O_2_ homeostasis-regulating cellular compartments containing peroxide-producing as well as peroxide-decomposing enzymes such as catalase (CAT). An increasing number of functions have been identified in plant peroxisomes, which contain more than 50 different enzymes (Hu et al., 2012; Reumann and Bartel, 2016; Kao et al., 2018).

Fatty acid β-oxidation is an important source of metabolic energy and carbohydrates. In higher plants, this peroxisomal pathway is linked to the glyoxylate cycle, which plays a particularly significant role in germinating seeds (Hu et al., 2012). Other essential peroxisomal plant functions include glycolate metabolism involved in photorespiration, hormone biosynthesis (auxins, jasmonic acid, and salicylic acid), and purine and polyamine catabolism (Sandalio and Romero-Puertas, 2015). Most of these processes give rise reactive oxygen species (ROS) production, whereas peroxisomes also produce nitric oxide (NO) and its derivative nitrogen reactive species (RNS) (Sandalio and Romero-Puertas, 2015). The great versatility of peroxisomes enables them to develop and share many metabolic and signaling pathways with other organelles, including chloroplasts and mitochondria, which frequently exchange metabolites and signals to coordinate their functions (Linka and Theodoulou, 2013; Shai et al., 2016).

Higher plants greatly depend on the regulation of biogenesis and recycling of available nutrients and energy sources to withstand and adapt to constant environmental changes. Despite their crucial role in rapid responses to these changes by synthesizing ROS and RNS, which are signaling molecules that regulate developmental processes and stress responses, many peroxisomes cause severe oxidative and nitrosative damage (Marinho et al., 2014; Mittler, 2017; Castillo et al., 2018). Peroxisomes experience highly dynamic, mostly ROS-regulated, changes in metabolism, size, morphology, velocity, and abundance depending on cell type, developmental stage, and environmental stimuli (Rodriguez-Serrano et al., 2016). Peroxisomal homeostasis, regulated by peroxisomal biogenesis and degradation, is essential for cell viability. Excess, damaged, and obsolete peroxisomes can be degraded by protease-dependent pathways or selective autophagy termed as pexophagy. Although much research has been devoted to deciphering pexophagy in yeast and mammal cells, less is known about plant pexophagy. In this review, we focus on the regulation of plant peroxisomal populations, with particular emphasis on the function and regulation of pexophagy under developmental and stress conditions.

### Peroxisome Formation and Proliferation

The regulation of peroxisome abundance is governed by the biogenesis, proliferation, and degradation of these tightly controlled organelles. Proteins involved in peroxisome biogenesis and its maintenance are called peroxins (PEXs) (Kao et al., 2018). Although these proteins have been well characterized in yeast and mammals, many questions regarding their role in plants remain unanswered.

In yeast, peroxisome biogenesis occurs according to two models: 1) *de novo* biogenesis from the endoplasmic reticulum (ER) and 2) directly through preexisting peroxisomes by growth and division (Aksit and van der Klei, 2018). Although not fully understood in plants, this process resembles that observed in mammals. Thus, as in mammals, PEX16 is delivered to peroxisomes from the ER and then recruits other peroxisomal membrane proteins (PMPs). The earlier acting PEX3, PEX16, and PEX19 directly insert PMPs into the peroxisomal membrane or a peroxisome-destined region of the ER (reviewed by Hu et al., 2012; Kao et al., 2018).

Although peroxisomes can divide by fission during cell division, they can also proliferate under various stress conditions. Excessive light (Desai and Hu, 2008), salinity (Mitsuya et al., 2011), ozone (Oksanen et al., 2003), H_2_O_2_ application (Lopez-Huertas et al., 2000), and xenobiotics, such as clofibrate (Castillo et al., 2008) and the heavy metal Cd (Rodriguez-Serrano et al., 2016), can rapidly increase plant peroxisome abundance. Peroxisome proliferation involves PEX11-regulated elongation and dynamin-related proteins and fission protein (FIS1)-regulated fission. Some evidence show that peroxisomal proliferation is regulated by ROS (Lopez-Huertas et al., 2000; Rodriguez-Serrano et al., 2016). Although peroxisome proliferation could be a protective response to stress conditions, its function and regulation are not fully understood.

### Peroxisome Degradation

As peroxisomes house several oxidative metabolic pathways, their proteins are prone to oxidative damage and thus require regulated turnover. Peroxisomes containing obsolete or dysfunctional proteins need to be eliminated to ensure that cellular redox homeostasis is maintained to avoid excessive ROS accumulation. These situations, which are particularly frequent under biotic and abiotic stress conditions, can also occur during cell differentiation or development and even under normal cellular conditions (Luo and Zhuang, 2018). Like other eukaryotes, through a process called autophagy, plants remove, degrade, and recycle damaged or unnecessary cell components and organelles (Avin-Wittenberg et al., 2018). There are several types of autophagy: microautophagy, macroautophagy, chaperone-mediated autophagy, and cytoplasm-to-vacuole transport, but so far only microautophagy and macroautophagy have been identified in plants (Zientara-Rytter and Sirko, 2014; Galluzzi et al., 2017; Avin-Wittenberg et al., 2018). Microautophagy involves a direct invagination of the tonoplast engulfing the cellular components to be degraded in the vacuole, whereas, in macroautophagy, the *de novo* formation of a double-membrane organelle, called autophagosome, engulfing these components is required. In plants, both microautophagy and macroautophagy (hereafter referred to as autophagy) can be selective or non-selective. Depending on the cargo, different types of selective autophagy have been reported: pexophagy (peroxisomes), mitophagy (mitochondria), chlorophagy (chloroplasts), reticulophagy (ER), and xenophagy (plant pathogens) (Till et al., 2012; Yoshimoto, 2012; Liu and Bassham, 2013; Zientara-Rytter and Sirko, 2014; Michaeli et al., 2016; Barros et al., 2017; Hafren et al., 2017; Izumi et al., 2017; Avin-Wittenberg et al., 2018).

A conserved set of proteins encoded by autophagy-related genes (ATGs) are involved in regulating autophagy in all eukaryotic cells and have also been reported in crop species (Yoshimoto, 2012; Young et al., 2019).

Unlike in other eukaryotes, more than 30 ATG proteins have been identified in plants, suggesting a higher diversity of gene families, probably due to their sessile properties. ATGs are organized in four main cores for autophagosome initiation and formation: 1) the ATG1 complex containing ATG1, ATG13, ATG11/17, and ATG101 for autophagosome initiation and formation; 2) the VPS34 complex containing ATG6, VPS34, VPS15, and ATG14; 3) the ATG9 complex containing ATG9, ATG18, and ATG2 for phagophore expansion; and 4) the ATG8 conjugation system containing ATG4, ATG8, ATG7, ATG10, ATG12, ATG16, and ATG5 (Avin-Wittenberg et al., 2018).

The ubiquitin-like protein ATG8, which binds the growing autophagosome membrane by covalently bonding to phosphatidylethanolamine, is essential for selective cargo recruitment and autophagosome-tonoplast fusion (Kellner et al., 2017; Avin-Wittenberg et al., 2018). Whereas yeast and fungi contain a single ATG8, higher eukaryotes have several ATG8 isoforms (nine in *Arabidopsis*), and it is unclear whether each isoform has a different function (Kellner et al., 2017). The residues forming W and L pockets of ATG8 are considerably conserved, whereas those neighboring the core pockets may determine ATG8 binding specificity (Kellner et al., 2017). Autophagy receptors bind ATG8 *via* an ATG8-interacting motif (AIM), currently referred to as the xLIR motif, with the consensus core sequence (ADEFGLPRSK)(DEGMSTV)(**WFY**)(DEILQTV)(ADEFHIKLMPSTV) (**ILV**), where the residues in positions 3 and 6 are the most crucial for interaction with ATG8 family proteins (Jacomin et al., 2016). Given this strong binding and evidence of ATG8 cross-kingdom conservation, this protein has been widely used to monitor autophagy (reviewed by Avin-Wittenberg et al., 2018; Young et al., 2019).

*Arabidopsis* ATG mutants have played a critical role in the study of pexophagy in plants, which was first reported in 2013 (Farmer et al., 2013; Kim et al., 2013; Shibata et al., 2013; Lee et al., 2014; Yoshimoto et al., 2014). By analyzing *Arabidopsis* mutants with peroxisomes abnormally clustered (*peups*) in leaves, Shibata et al. (2013) found that ATG8 proteins were localized on the surface of peroxisomes, with ATG2, ATG18a, and ATG7 proteins involved in specific peroxisome degradation processes. *Arabidopsis* mutants in these genes contain high levels of insoluble inactive CAT (Shibata et al., 2013). Recently, peroxisome accumulation was observed in *atg5* and *atg7* mutants in leaves from *Arabidopsis* plants exposed to Cd, whereas phagophores surrounding peroxisomes were observed in wild-type (WT) *Arabidopsis* plants expressing peroxisomal markers ATG8a-GFP and SKL-CFP (Calero-Muñoz et al., 2019). The application of exogenous H_2_O_2_ produces peroxisome clustering, suggesting that H_2_O_2_-induced oxidative damage in peroxisomal proteins could signal pexophagy induction (Shibata et al., 2013). However, although the role of CAT in autophagy induction has been under debate, the analysis of CAT-defective mutants has shown that CAT is dispensable for peroxisome degradation (Shibata et al., 2013). Some evidence suggest that CAT activity is involved in ROS-regulated autophagy-dependent programmed cell death induced by hydroxyurea and *avrRpm1* (Hackenberg et al., 2013) and starvation-induced pexophagy (Tyutereva et al., 2017). Calero-Muñoz et al. (2019) showed that ATG8a co-localizes with CAT in the electron-dense peroxisomal core where inactive CAT is accumulated. Further studies of *Arabidopsis* ATG mutants have revealed that clustered peroxisomes mainly accumulate in the aerial parts of plants, where oxidative pathways, mainly β-oxidation and photorespiration, take place rather than in roots (Yoshimoto et al., 2014). However, pexophagy has recently been reported to be also involved in peroxisomal quality control in *Arabidopsis* root meristems (Huang et al., 2019).

### Pexophagy Receptors

The selective autophagy pathways in eukaryotes require specific cargo receptor(s) and/or adaptors. Two kinds of autophagy cargo receptor have been described in yeast and mammals, which differ in their capacity to bind ubiquitylated cargos (Zientara-Rytter and Sirko, 2016). Four pexophagy receptors/adaptors have been reported in eukaryotes: the yeast Atg36 (*Saccharomyces cerevisiae*) and Atg30 (*Pichia pastoris*), unable to bind ubiquitin, and the mammalian NBR1 (neighbor of Brca1 gene) and p62, which recognize ubiquitinated proteins (Zientara-Rytter and Sirko, 2016). ScAtg36 interacts with Pex3 PMPs as well as with Atg8 and Atg11 *via* AIM and Atg11-binding site, respectively (Farre et al., 2013; Honsho et al., 2016). PpAtg30 requires both Atg37 and Pex3 to recruit Atg11 and Atg8 to the pexophagic receptor-protein complex (Zientara-Rytter et al., 2018). PpAtg30 also interacts with Pex14 (Farre et al., 2008), although only phosphorylated PEX14 is involved in pexophagy in *Hansenula polymorpha* (van Zutphen et al., 2008). No homologs of Atg30 or Atg36 are known in plants. However, plants have NBR1-like protein (NBR1 from *Arabidopsis* and Joka2 from tobacco), which is a functional hybrid of the two mammalian ubiquitin-binding autophagy receptors, NBR1 and p62, required for targeting ubiquitinated peroxisomes to autophagosomes for degradation (Deosaran et al., 2013; Walter et al., 2014; Yamashita et al., 2014). However, its role in pexophagy needs further investigation (Zhou et al., 2013; Hafren et al., 2017; Young et al., 2019). The co-localization of NBR1 and ATG8 in electron-dense peroxisomal cores in *Arabidopsis* plants exposed to Cd has recently been reported (Calero-Muñoz et al., 2019). However, using forward genetic screening, which consistently recovers *Arabidopsis atg* mutations by exploiting LON2 (peroxisomal matrix protease)-defective mutants (*lon2*), Young et al. (2019) showed that lon2 peroxisome autophagy does not require NBR1, which, however, may play an important role in LON2-independent pexophagy (Young et al., 2019). As reported in yeast and mammals, the ubiquitination of PEXs, such as PEX3, PEX5, and PEX14, may be also involved in plant pexophagy (Michaeli et al., 2016; Young and Bartel, 2016). Interestingly, CAT is ubiquitinated and preferentially accumulated in *Arabidopsis nbr1* mutants, which support possible NBR1/CAT interactions (Zhou et al., 2013). Dominant suppressor of KAR2 (DSK2) is another ubiquitin receptor candidate in plants, which binds the transcription factor BES1 (BRI1-EMS-suppressor 1) for its autophagic degradation (Nolan et al., 2017). In *Arabidopsis*, DSK2 interacts with the ring PEX2 and PEX12, which act as E3 ligases (Kaur et al., 2013). However, PEXs could also interact directly with ATG8 without involving a bridging receptor. Using bioinformatics and bimolecular fluorescence complementation techniques, Xie et al. (2016) found that PEX6 and PEX10 may interact with ATG8. However, Gonzalez et al. (2018) have reported that the physiological defects of *pex6-1* in *Arabidopsis* mutants are partially restored by preventing autophagy (disabling ATG7), which would rule out a role for PEX6 in promoting autophagy.

Although the signals determining plant peroxisome degradation have not been fully identified, ROS and oxidative stress factors, such as CAT and peroxisomal oxidation, are commonly cited (Shibata et al., 2013; Yoshimoto et al., 2014; Calero-Muñoz et al., 2019). Moreover, *Arabidopsis atg* mutants are more hypersensitive to oxidative stress, suggesting that pexophagy could be an important component in oxidative stress responses (Zhou et al., 2013). In mammalian cells, ROS induce pexophagy by promoting PEX5 phosphorylation and ubiquitination (Zhang et al., 2015). Although several PEXs have been reported to be phosphorylated in *Arabidopsis* (Kataya et al., 2019), a direct relationship between ROS, PEX phosphorylation and pexophagy has not been established.

ROS induce chloroplast extensions (stromules), which are associated with chlorophagy involving Rubisco-containing bodies (Hanson and Hines, 2018). Similar ROS-dependent extensions (peroxules) have been observed in peroxisomes under stress conditions (Sinclair et al., 2009; Jaipargas et al., 2016; Rodriguez-Serrano et al., 2016), although no evidence of their role in vesicle formation to recycle peroxisomal components has been reported.

Cysteine-generated sulfide in the cytosol negatively regulates and inhibits autophagy in *Arabidopsis* plants by preventing ATG8 accumulation (Alvarez et al., 2012) probably through the persulfidation of enzymes involved in autophagosome formation (Alvarez et al., 2012). Although sulfide has not been shown to affect pexophagy, as some persulfidated peroxisomal proteins have been reported, most of which are associated with antioxidant defenses (Aroca et al., 2017), persulfidation could therefore be involved in pexophagy regulation.

### Pexophagy and Peroxisomal Metabolic Transition

**Figure 1:**
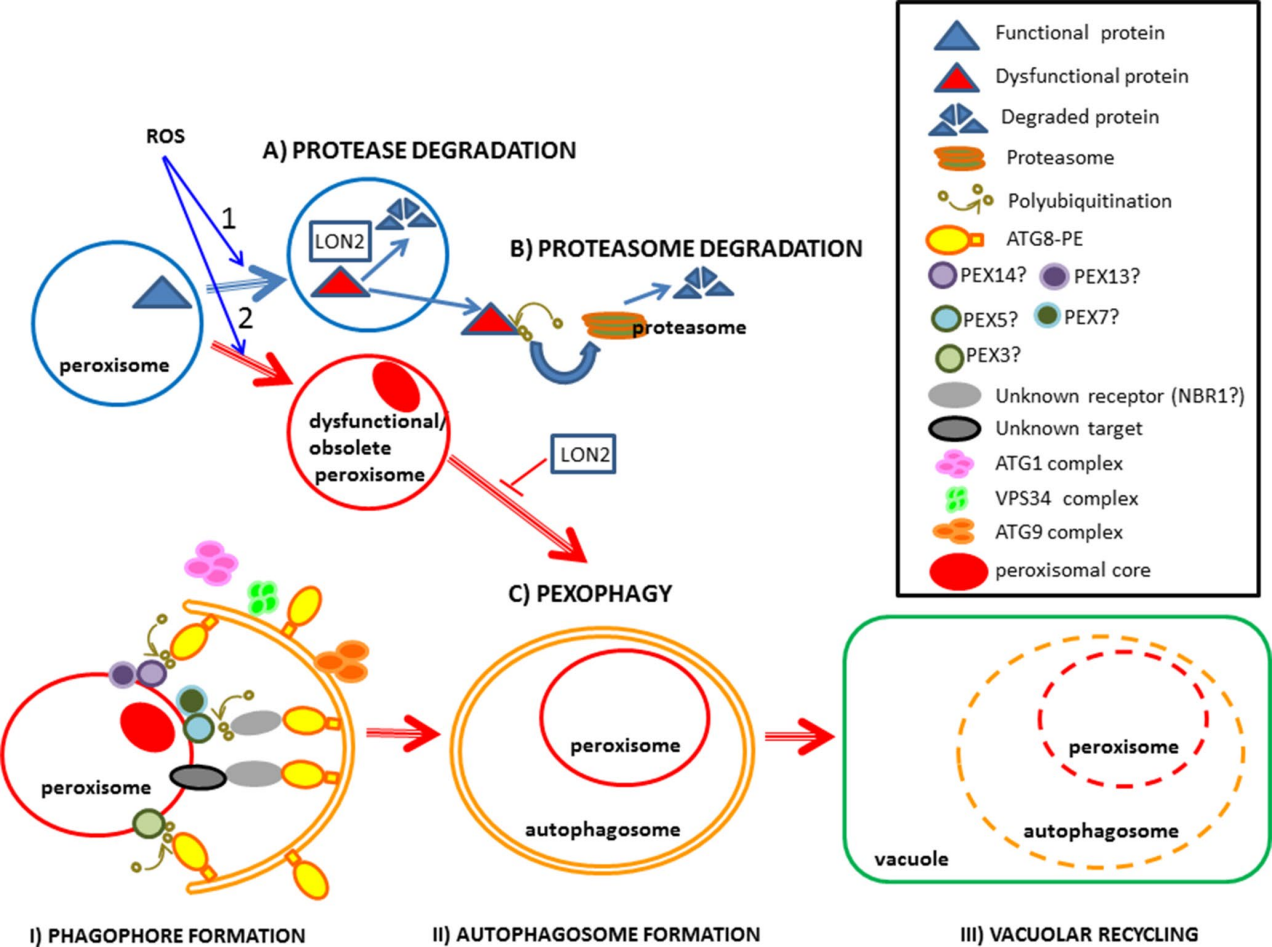
Diagram illustrating the elimination of peroxisomal proteins and obsolete/dysfunctional peroxisomes. 1) Peroxisomal proteins can be eliminated by: **(A)** endogenous peroxisomal proteases such as LON2 or **(B)** through proteasome degradation in the cytoplasm following ubiquitination. 2) Peroxisomes can be eliminated by: **(C)** pexophagy, involving three main steps: I) the formation of phagophores when they begin to surround the recyclable peroxisome; II) autophagosome formation when the peroxisome is completely engulfed in the autophagosome and III) vacuolar recycling when the peroxisome inside the vacuole starts to degrade. ROS produced during developmental processes, starvation and biotic or abiotic stresses induce the elimination of peroxisomal proteins, as well as obsolete and dysfuncional peroxisomes. Pexophagy components followed by a question mark in the diagram, whose precise role in plants remains unclear, require further study.

During light-induced seed germination and seedling establishment, the transition from glyoxysome, which are present in seeds involved in the glyoxylate cycle and β-oxidation, to leaf-type peroxisomes containing photorespiration machinery is triggered. This transition degrades expendable glyoxylate cycle enzymes such as isocitrate lyase (ICL) and malate synthase (MLS), whereas photorespiration-related enzymes are imported into peroxisomes. Farmer et al. (2013) reported that peroxisomal protease LON2 plays an important role in the selective degradation of obsolete matrix proteins during this metabolic transition (**Figure 1**). These proteins are also exported to the cytoplasm and degraded by proteasome after ubiquitination (**Figure 1**). LON2 proteins are a conserved family of ATPases characterized by chaperone and protease activity (Goto-Yamada et al., 2014; Young and Bartel, 2016). *Arabidopsis* mutants expressing protease-deficient AAA-active LON2 in a *lon2*-null genetic background have lower pexophagy levels, demonstrating that LON2 chaperone activity inhibits pexophagy (Farmer et al., 2013; Young and Bartel, 2016). Stabilization studies of *atg* and *lon2* mutants showed that both LON2 and autophagy affect ICL and MLS turnover, whereas pexophagy mainly affects the turnover of thiolase involved in β-oxidation (Young et al., 2019), suggesting that different pathways control peroxisomal components.

### Pexophagic Regulation of Peroxisomal Quality

Pexophagy mediates peroxisome turnover even under non-stress conditions, as demonstrated by peroxisome clustering in *Arabidopsis atg* mutants, which may regulate basal peroxisome levels, mainly in green tissues, where H_2_O_2_-producing photorespiration is highly active (Farmer et al., 2013; Kim et al., 2013; Shibata et al., 2013; Lee et al., 2014; Yoshimoto et al., 2014). Although *Arabidopsis atg* mutants accumulate more peroxisomes than their WT counterparts, this is not the case for other organelles such as the Golgi apparatus, mitochondria, ER, and chloroplasts. Maize *atg* mutants also accumulate PEX14, a peroxisomal marker (Li et al., 2015). In *Arabidopsis atg5* mutants, analyses of peroxisomal redox status have shown a larger number of oxidized organelles than in WT plants (Yoshimoto et al., 2014). All these findings are consistent with the possibility that pexophagy clears damaged peroxisomes at a basal rate in the absence of stresses typically associated with autophagy induction (Yoshimoto et al., 2014).

### Pexophagy, Plant Development, and Responses to Stress

Root growth and architecture are controlled by meristems, where stem cell activity is regulated by auxins, nutrient availability, and ROS homeostasis (Rellan-Alvarez et al., 2016). Pexophagy plays a key role in the glucose-mediated regulation of root meristem activity by maintaining ROS and auxin cellular homeostasis in *Arabidopsis* plants (Huang et al., 2019). Using yeast two-hybrid and co-immunoprecipitation assays, Huang et al. (2019) observed interactions between ATG8e and the peroxisomal ATP-binding cassette D1 (ABCD1) Walker B motif and that the glucose-tolerant root elongation phenotype of *atg5* and *atg7* mutants is partially rescued by ABCD1 overexpression.

Biochemical, cytological, and pharmacological analyses have provided evidence of peroxisome degradation by autophagy during carbohydrate starvation in *Nicotiana tabacum* cells, which is slowed down by the application of autophagy inhibitor 3-methyladenine (Voitsekhovskaja et al., 2014). Exposure to Cd induces peroxisome oxidation and pexophagy, whereas the reduction of ROS production in *Arabidopsis* mutants *gox2* (glycolate oxidase 2) and *rbohC* (NADPH oxidase C) considerably reduces this process. Pexophagy is an important component in rapid plant responses to Cd, which prevents disturbances in peroxisomal populations and the cell redox balance (Calero-Muñoz et al., 2019).

## Future Perspectives

Plants develop complex signaling mechanisms to adapt to and withstand constant environmental changes. Many cell/organelle cross-talk mechanisms are fine tuned by ROS. The regulation of peroxisome populations, which, in turn, affects peroxisomal metabolism and redox homeostasis, is essential for plant life. Although plant pexophagy is currently the subject of intense study, important aspects of this complex process remain unexplained. Questions regarding how, why, and when plants regulate peroxisome populations, the possible adaptive role of peroxisome proliferation in plants, and how exactly pexophagy is initiated and terminated remain unanswered. Little is known about the effect of other molecules, such as H_2_S, and NO, on pexophagy. Further studies are required to pinpoint the receptors that connect the peroxisome to be degraded to pexophagic machinery and to identify possible targets in the peroxisome to be recognized by putative receptor(s). Finally, the mechanisms enabling plants to induce and regulate pexophagy at a basal level also need to be deciphered.

## Author Contributions

The manuscript was prepared by AO and reviewed and rewritten by LMS.

## Funding

This study was co-funded by ERDF grant BIO2015-67657-P and PGC2018-098372-B-I00 from MICINN, I-LINK1247 from CSIC, and TRANSAUTOPHAGY EU Framework Program Horizon 2020 COST grant OC-2015-1-19840. The authors wish to apologize to those colleagues whose work has not been cited due to space limitations. The authors wish to thank Michael O’Shea for proofreading the manuscript.

## Conflict of Interest Statement

The handling editor declared a past co-authorship with one of the authors LS.
